# Treatment with FTY720 has no beneficial effects on short-term outcome in an experimental model of intracerebral hemorrhage

**DOI:** 10.1186/s13231-016-0016-z

**Published:** 2016-02-18

**Authors:** Frieder Schlunk, Waltraud Pfeilschifter, Kazim Yigitkanli, Eng H. Lo, Christian Foerch

**Affiliations:** Department of Neurology, Johann Wolfgang Goethe-University, Schleusenweg 2-16, 60528 Frankfurt, Germany; Neuroprotection Research Laboratory, Massachusetts General Hospital, Harvard Medical School, Charlestown, MA USA

**Keywords:** Intracerebral hemorrhage, FTY720, Animal model, Edema, Blood–Brain-Barrier

## Abstract

**Background:**

No evidence-based therapy is available for patients with acute intracerebral hemorrhage (ICH). In view of the profound inflammatory reaction in the perilesional tissue, we investigated in a well-characterized experimental model whether the administration of the immunomodulator fingolimod (FTY720) is neuroprotective in acute ICH.

**Methods:**

ICH was induced by means of a stereotactic intrastriatal injection of collagenase type VII-S. FTY720 (1 mg/kg) was administered intraperitoneally 1 h after ICH induction. Hematoma volume was assessed spectrophotometrically at 24 h after ICH induction. The following endpoints were determined at 24 and 72 h, respectively: mortality rate and neurologic outcomes, edema formation, and MMP-9 activity.

**Results:**

Twenty-four hour after ICH induction, hematoma volume was not statistically different between groups. No difference was found in mortality and neurologic outcomes at 24 and 72 h between FTY720 treated mice and controls. Edema formation was present in both groups on the ipsilateral side with no statistical difference between groups at both time points. No difference was found in MMP-9 levels after 24 and 72 h between groups.

**Conclusions:**

Our results suggest that FTY720 has no beneficial effects in the acute phase of experimental ICH.

**Electronic supplementary material:**

The online version of this article (doi:10.1186/s13231-016-0016-z) contains supplementary material, which is available to authorized users.

## Background

Intracerebral hemorrhage (ICH) accounts for approximately 15 % of all strokes worldwide. It is a severe disease with high mortality rates and poor functional outcomes [1]. A primary phase of “mechanical” brain damage induced by the expanding hematoma can be distinguished from a phase of secondary injury mediated by thrombin, free iron radicals, and inflammation [2]. In fact, leukocyte invasion into the brain starts immediately after the bleeding has occurred [3]. Inflammation contributes to the disintegration of the blood–brain-barrier (BBB) and thus facilitates brain edema formation [2]. Besides neutrophils, CD3 positive lymphocytes have recently been identified in the perihematomal region in experimental ICH [4].

Fingolimod (FTY720) is an immune-modulating drug that has been approved for the treatment of patients with relapsing-remitting multiple sclerosis. Its anti-inflammatory effect is mediated by agonist-induced internalization of S1P1-receptors on T-lymphocytes, which inhibits lymphocyte egress from primary and secondary lymphoid organs [4, 5]. Several experimental studies have shown a protective effect of FTY720 therapy in ischemic stroke in terms of reducing lesion size and cell death [5–9].

Considering the strong inflammatory response that follows hematoma formation, we speculated that FTY720 treatment could be beneficial in the acute phase of ICH. Using a reliable animal model [10–13] and a well-powered study design, we investigated mortality, short-term functional outcome, MMP-9 activation and edema formation in mice treated with FTY720 or placebo.

## Methods

### Animals

All experiments were performed in accordance with the guide from the National Institute of Health for the care and use of laboratory animals. Throughout this study, male CD1-mice aged 12–16 weeks were used.

### Study design

**Fig. 1:**
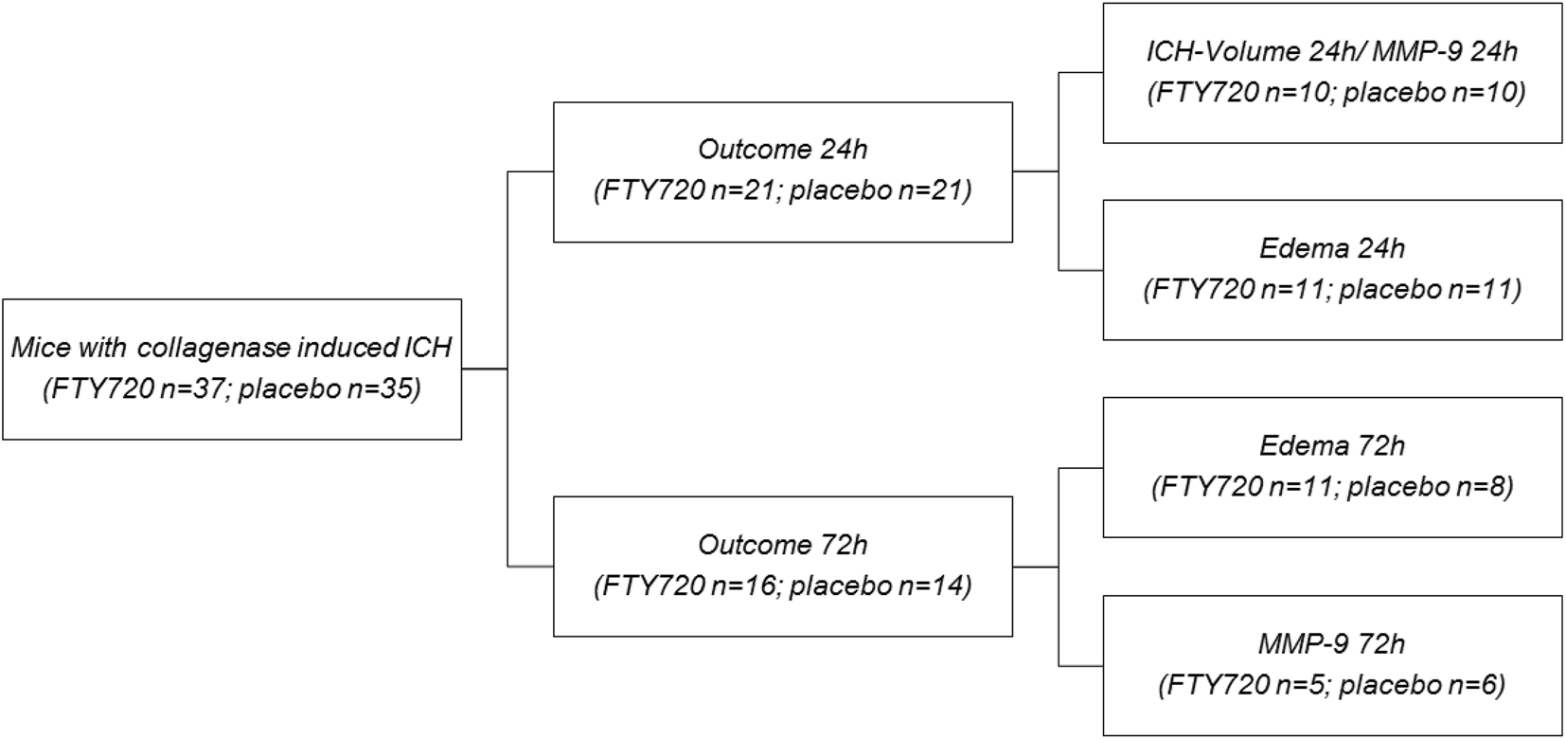
Experimental design

An overview of the experimental design is given in Fig. 1. Animals were randomly assigned to receive FTY720 or vehicle before any study procedures were undertaken. Endpoints were measured at two different time points (24 h and 72 h respectively). Group sizes were determined based on our previous studies using this model where we demonstrated the effect of anticoagulation and anticoagulation reversal on hematoma growth [10, 14–16]. A first set of animals was used to determine hematoma volume and MMP-9 activity levels 24 h after ICH induction [n = 20 mice, randomized to either FTY720 treatment (n = 10) or placebo (n = 10)]. A second and third set was used to investigate edema formation at 24 h [n = 22 mice, randomized to either FTY720 treatment (n = 11) or placebo (n = 11)] or at 72 h [n = 19 mice, randomized to either FTY720 treatment (n = 11) or placebo (n = 8)], respectively. A fourth set of mice was used to determine MMP-9 levels at 72 h [n = 11 mice, randomized to either FTY treatment (n = 5) or placebo (n = 6)]. Functional outcome was investigated on all mice from the upper mentioned groups at 24 h in order to optimize group sizes for non-parametric testing [i.e., n = 42; FTY720 (n = 21), placebo (n = 21)] and at 72 h [i.e., n = 30; FTY720 (n = 16), placebo (n = 14)], respectively.

### Drug administration/white blood cell count

**Fig. 2:**
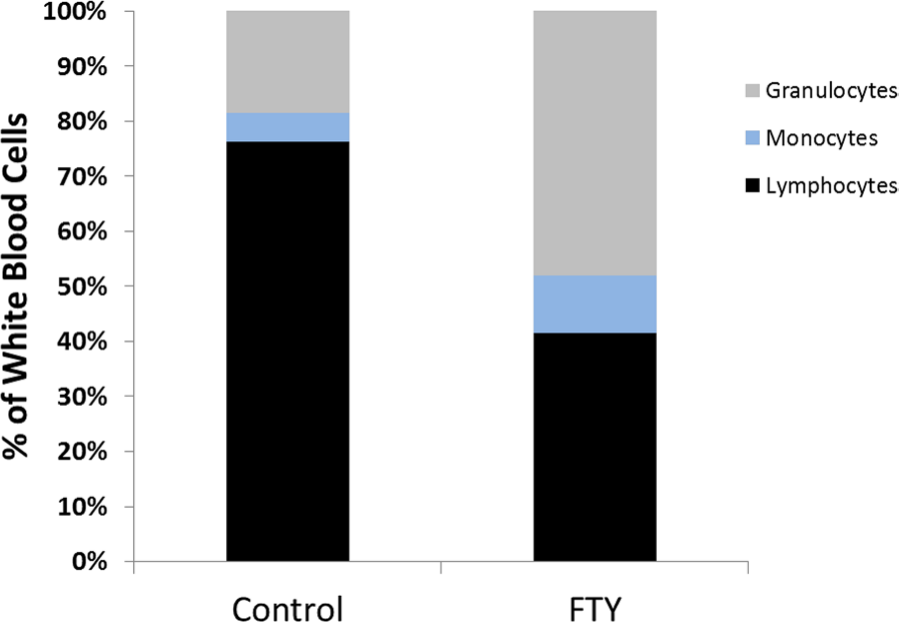
White blood cell count. FTY720 or saline were injected into otherwise untreated mice. Twenty-four hours after injection a white blood cell count was performed. Mean percentage of lymphocytes in saline or FTY720 treated mice was 76.3 % and 41.5 %, respectively (n = 2 per group)

FTY720 (1 mg/kg body weight, dissolved in 0.9 % saline) or an equal amount of saline was administered via intraperitoneal injection in a blinded fashion 1 h after ICH induction. This FTY720 dosing regime has been proven to reduce the number of circulating lymphocytes and to provide neuroprotection in several experimental studies in ischemic stroke [5, 6]. To crudely demonstrate the effects of FTY720 or saline injection on the immune system in our model, FTY720 or saline were injected into otherwise untreated mice. Twenty-four hours after injection, 700 µL of blood were drawn from the inferior caval vein under deep anesthesia with a 19-Gauge needle and transferred into EDTA microtainer tubes. A white blood cell count was performed on a fully automated analyzer at the Massachusetts General Hospital routine laboratory and results were confirmed by manual blood film review. Mean percentage of lymphocytes of saline or FTY720 was 76.3 and 41.5 %, respectively (n = 2 per group). Mice that received FTY720 injection showed a marked shift towards a higher fraction of granulocytes (Fig. 2).

### ICH induction

ICH was induced by means of a stereotactic injection of collagenase type VII-S (Sigma Aldrich, St. Louis, MO). For doing so, mice were anesthetized with isoflurane (1.5–2 %) in a 70 % N_2_O- and 30 % O_2_-mixture and placed in a stereotactic frame. A small borehole was drilled in the skull. A 32-Gauge needle (Hamilton 7000 series, Hamilton, Reno, NV) was lowered into the right striatum at the following coordinates from the bregma: 0.0 mm anterior, 2 mm lateral and 3.5 mm in depth. Over a period of 5 min, collagenase (0.1 U in 0.5 µL saline) was injected. The needle was left in situ for 10 min and was then slowly withdrawn over 5 min. After suturing, the animals were allowed to wake up and returned to their cages. Throughout the procedure, a heating lamp was used to maintain body temperature.

### Functional outcome

Functional outcome was assessed at 24 and 72 h after ICH induction, respectively, by means of an established ordinal neuroscore and a standard hanging wire test [17]. For determining the neuroscore, mice were placed on a plate. Spontaneous movements were observed for 1 min and categorized as follows: 0 = no apparent deficit; 1 = slight deficit as instability during walking, but no circling; 2 = circling to the right with at least some straight movements and covering of distance; 3 = heavy circling to the right, no gain of distance or no movements at all. Dead animals were categorized as 4. For the hanging wire test, mice were placed on a wire approximately 30 cm above the surface, and the time to falloff was measured. A maximum of 60 s was allowed and the test was repeated three times.

### Hematoma volume

Hematoma volume was quantified 24 h after collagenase injection. Briefly, the coagulated hematoma was carefully separated from the perilesional tissue on 1 mm brain slices using a scalpel and was transferred into a glass tube containing 3 ml of PBS. After homogenization, ultrasound was applied to lyse erythrocyte membranes. The samples were then centrifuged, and 250 µL of the supernatant was transferred into 1000 µL of Drabkins reagent. Absorption was determined in a spectrophotometer at 540 nm, and the results were expressed in µL of blood on the basis of a standard curve.

### Brain edema measurements

To measure perilesional edema formation, mice were deeply anesthetized (isoflurane 5 %) and euthanized at 24 and 72 h after collagenase injection, respectively. After removing the brain, a 3 mm block was cut with the needle insertion point in the middle using a mouse brain matrix and divided into left and right hemisphere. The striatum (and the hematoma on the ipsilateral side) was removed, and the cortex was weighed on aluminum foil (of known weight) with an electronic scale in order to obtain the wet weight. The samples were then dried for 48 h at 80 °C and weighed again. Brain water content was expressed in mean percentage as followed: (wet weight - dry weight)/wet weight × 100. The cerebellum served as a control [18].

### Brain MMP-9 measurement

To determine MMP-9 activity, mice were deeply anesthetized at 24 or 72 h after ICH induction and transcardially perfused with PBS. Brain specimens were obtained, and three 1 mm slices were cut using a 1 mm mouse brain matrix. Slices were divided into left and right hemisphere. The hematoma in the right striatum was carefully removed with a scalpel leaving no visible traces of blood. The cortex specimen were transferred into Eppendorf tubes and frozen at −80 °C. For MMP-9 measurements, the contra- and ipsilateral cortex specimens were homogenized in 600 µL cell lysis buffer including protease inhibitors on ice. Thereafter, the samples were centrifuged for 30 min with 13,000 rpm at 4 °C. Protein concentration of the supernatant was measured by means of the Bradford assay (Bio-Rad). According to the protein concentration, samples were prepared, loaded and separated by 10 % Tris–glycine gel with 0.1 % gelatin as substrate. After electrophoretic separation, the gel was renaturated and then incubated with developing buffer at 37 °C for 20 h. After developing, the gel was stained with 0.5 % Coomassie blue R-250 and then destained appropriately. MMP activity was quantified using an ImageJ tool via determination of the integrated density.

### Statistical analysis

We used SPSS version 20 for statistical analyses. Parametric data were compared using the *t* test. Non-parametric data were compared by means of the Mann–Whitney-U test or the Chi square-test. p < 0.05 was considered as significant.

## Results

### Mortality and functional outcome

**Fig. 3:**
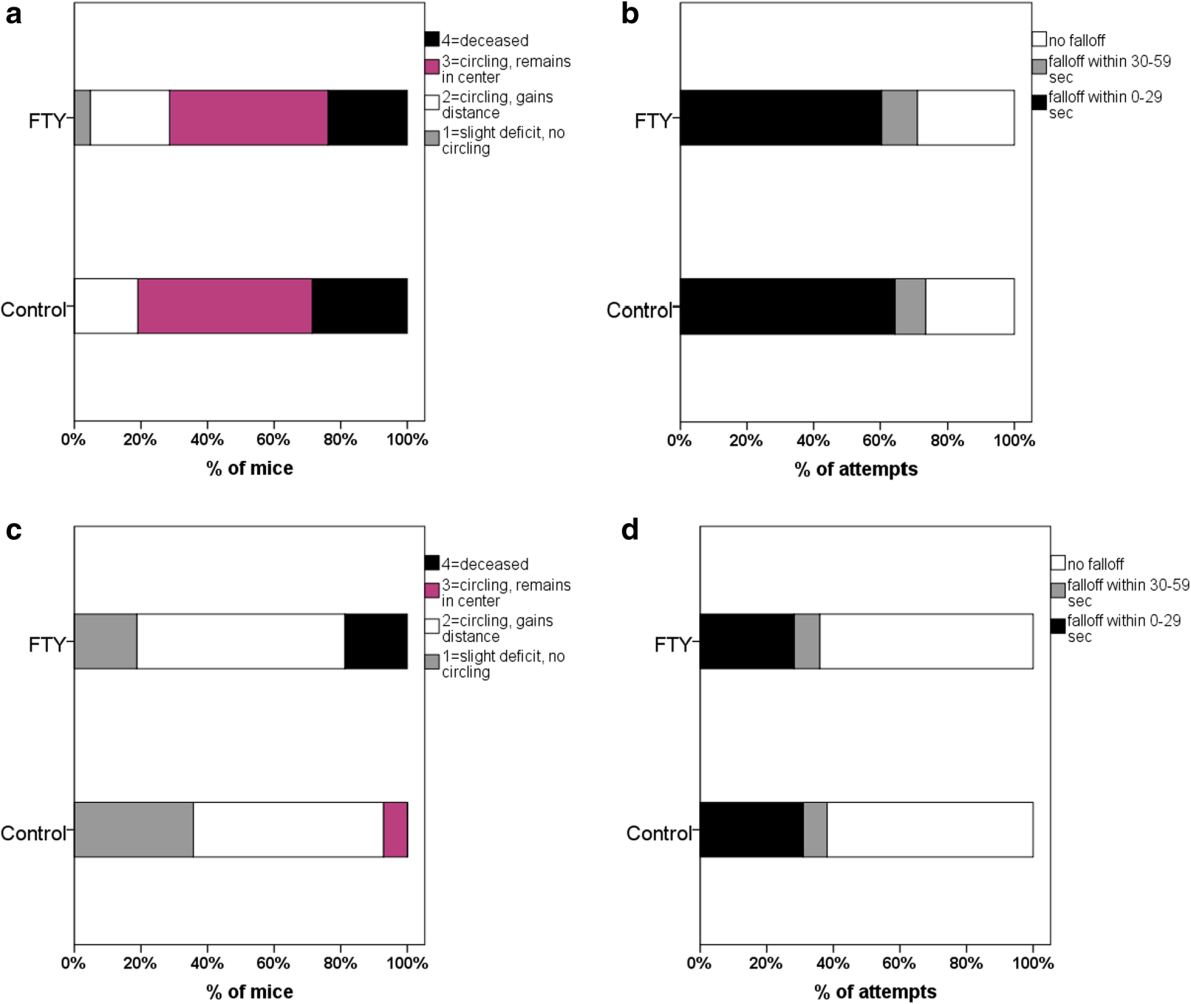
Outcome 24 and 72 h after ICH induction. A 5-point score and a standard hanging wire test were used. No statistical difference was found between FTY720 treated mice and controls at 24 h using the 5-point-score [FTY720: n = 21, controls: n = 21; p = 0.486 (**a**)] or the standard hanging wire test [p = 0.710 (**b**)]. Similarly, evaluation 72 h after ICH induction did not reveal a significant difference between groups using the 5-point score [FTY720: n = 16, controls: n = 14; p = 0.191 (**c**)] or the hanging wire test [p = 0.816 (**d**)]

At 24 h, mortality rate was 5/21 FTY720 treated mice and 6/21 control mice (p = 0.726). Neuroscores of FTY720 treated mice and control mice were found to be not different (p = 0.486; Fig. 3a). Regarding the results of the hanging wire test, there was also no indication of a possible protective effect of FTY720 treatment as compared to the placebo treatment (p = 0.710; Fig. 3b).

Seventy-two hour after ICH induction, mortality rate was 3/16 FTY720 treated mice and 0/14 control mice (p = 0.088). Functional outcome as measured on the neuroscore was again similar between FTY720 treated mice and controls (p = 0.191, Fig. 3c). Furthermore, no significant difference was found for the hanging wire test between groups (p = 0.816; Fig. 3d).

### Hematoma volume

**Fig. 4:**
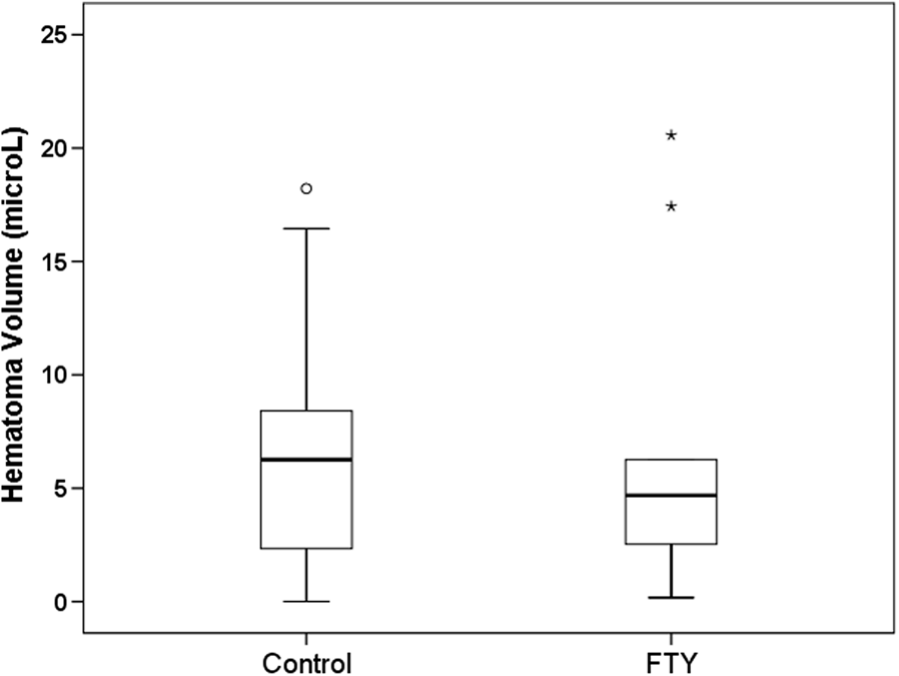
Hematoma volume 24 h after ICH induction. Quantitative hemoglobin measurement in FTY720 treated animals (n = 10) and controls (n = 10) revealed no difference between groups (p = 0.867). *Circles* denote outliers that are more than 1.5 interquartile ranges away from the nearer edge of the* box*. *Stars* denote outliers that are more than 3 interquartile ranges away from the nearer edge of the box

Hematoma volume was determined at 24 h after ICH induction. Mortality rate was 2/10 in the FTY720 treated group and 2/10 in the control group. Dead mice were included in the analysis. Mean hematoma volume was 7.1 ± 1.9 µL in control mice (n = 10) and 6.6 ± 2.2 µL in mice that received FTY720 injection (n = 10). There was no statistically significant difference between groups (p = 0.867; Fig. 4).

### Brain edema formation

Edema measurements were performed at 24 h and at 72 h after collagenase injection, respectively. Mice that died within these observation periods were excluded from the analysis, as edema measurements cannot be reliably performed in autolytic tissue.

**Fig. 5:**
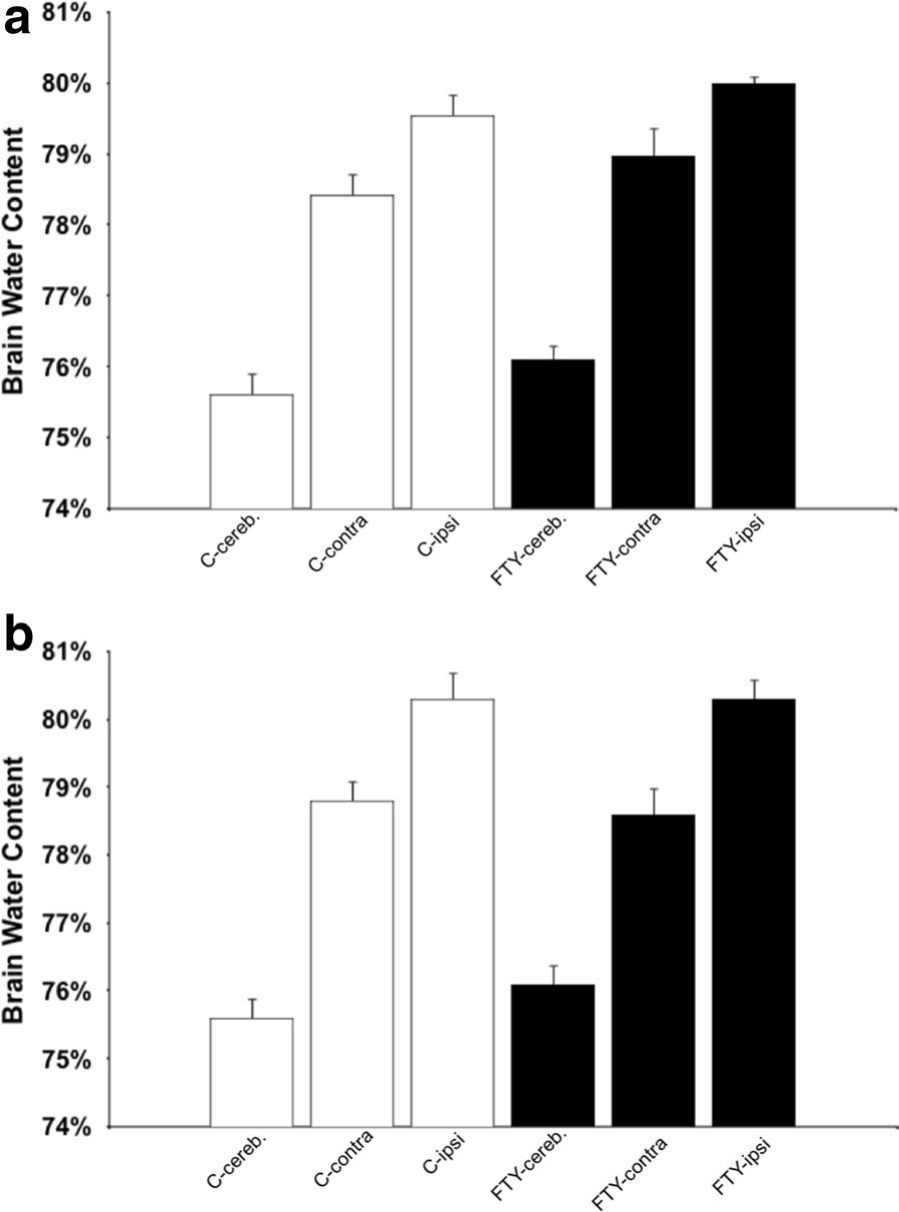
Edema formation 24 (**a**) and 72 h (**b**) after ICH Induction: Edema was assessed by means of the wet dry method in ipsi- and contralateral hemispheres. The cerebellum served as a control. Twenty-four hour after ICH induction, there was no statistical difference between ipsilateral hemispheres of FTY720 mice (n = 8) and controls (n = 7; p = 0.122). Similarly, at 72 h, no difference was found between groups (FTY720: n = 8, controls: n = 7; p = 0.914)

After 24 h, mortality was 3/11 in the FTY treated group and 4/11 in the control group. Mean brain water content was 78.4 ± 0.3 % on the contralateral and 79.5 ± 0.3 % on the ipsilateral side in the control group (n = 7; p = 0.018). In FTY720 treated mice, contralateral hemispheres showed 78.9 ± 0.4 % and ipsilateral hemispheres 80 ± 0.1 % of brain water content (n = 8; p = 0.033). No statistically significant difference was found between the ipsilateral sides among groups (p = 0.122; Fig. 5a).

Seventy-two hour after ICH induction, mortality rate was 3/11 in FTY720 treated mice and 0/8 in saline treated control mice. One mouse in the saline treated group died after outcome assessment but shortly before edema measurements and was therefore also excluded. Saline treated mice showed brain water contents of 78.8 ± 0.3 % on the contralateral side, and of 80.3 ± 0.4 % on the ipsilateral side (n = 7; p = 0.013). In the FTY720 treated group, brain water content was 78.6 ± 0.4 % on the contralateral side, and 80.3 ± 0.3 % on the ipsilateral side (n = 8; p = 0.005). Again, no significant difference was found between the ipsilateral sides among groups (p = 0.914; Fig. 5b).

### Brain MMP-9 measurements

MMP-9 activation was measured at 24 and at 72 h after ICH-induction, respectively. Mice that died within the observation period were excluded from this analysis. Specimens for MMP-9 measurements at 24 h were the same as were used for hematoma volume determination (see above).

**Fig. 6:**
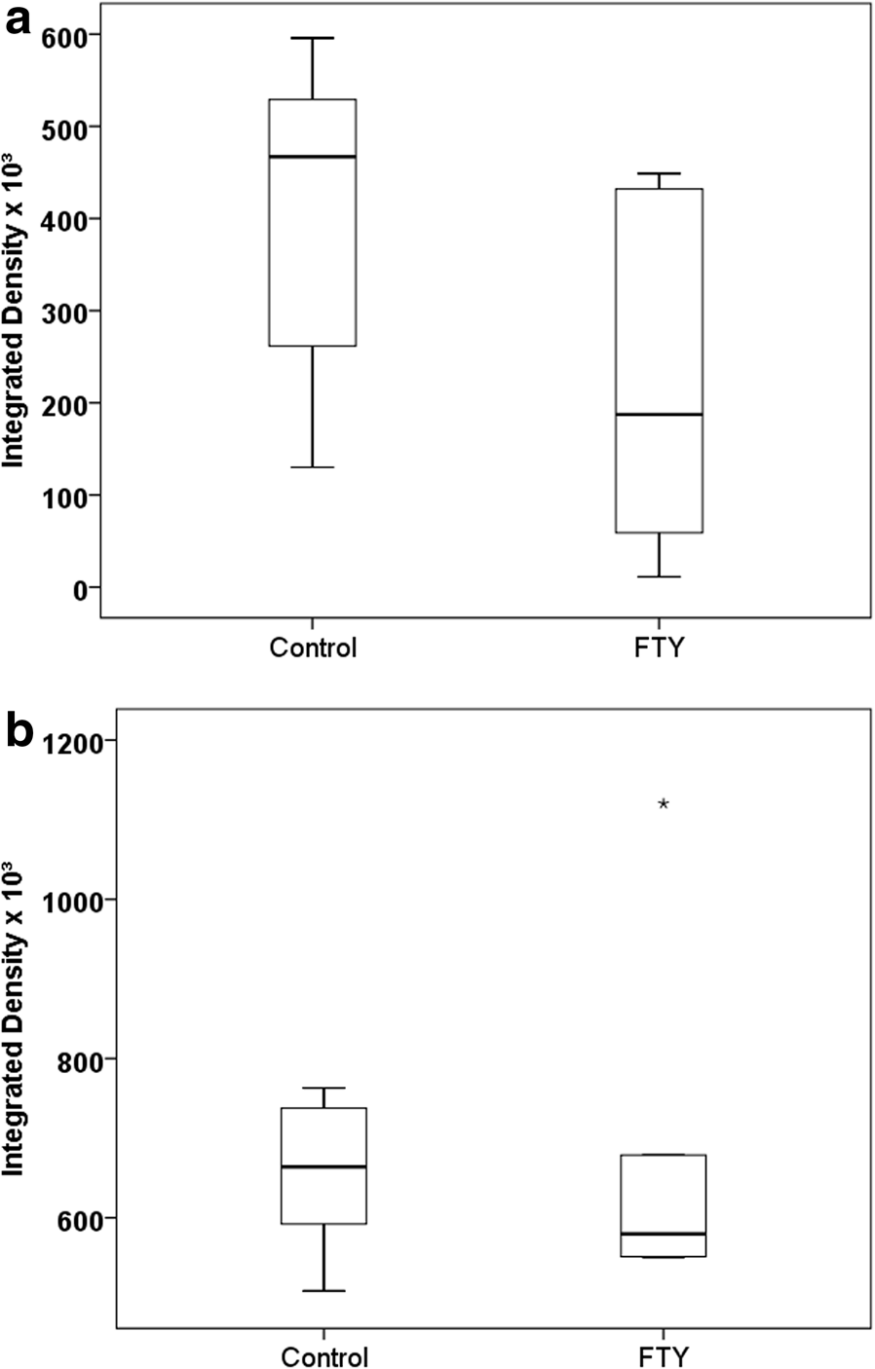
MMP-9 levels in gelatin zymograms at 24 (**a**) and 72 h (**b**) after ICH induction: The* graphs* show mean values of integrated density. Twenty-four hour after ICH induction there was a lower ipsilateral signal in FTY720 treated animals (n = 8) as compared to controls (n = 8) with a trend towards significance (p = 0.069). At 72 h, no difference was found for the ipsilateral side between FTY720 treated mice (n = 5) and saline treated controls (n = 6; p = 0.708)

Twenty-four hour after ICH induction, there was a trend towards a significant difference between groups (p = 0.069; Fig. 6a), with lower MMP-9 activity signals in FTY720 treated animals as compared to controls [assessed by means of the integrated density being 405.2 × 10^−3^ ± 59.3 × 10^−3^ on the ipsilateral side of saline treated mice (n = 8) and 227.1 × 10^−3^ ± 68.5 × 10^−3^ on the ipsilateral side of FTY720 treated animals (n = 8)].

Seventy-two hour after ICH induction, mortality rate was 0/5 in FTY720 treated animals and 0/6 in controls. Differences in MMP-9 activity levels were not confirmed at 72 h after bleeding induction (saline ipsilateral: 654.8 × 10^−3^ ± 38.4 × 10^−3^, n = 6; FTY720 ipsilateral: 696 × 10^−3^ ± 108.6 × 10^−3^, n = 5; p = 0.708; Fig. 6b; Additional file 1: Figure S1). As compared to the ipsilateral side, visual examination of gels with samples of the contralateral hemispheres showed clearly less MMP-9 signal at both time points in all specimens.

## Discussion

In our study, we analyzed whether FTY720 treatment provides short-term neuroprotection in acute ICH. Although our study was adequately powered and based on a well-defined and reliable experimental model of ICH, we were not able to demonstrate any differences in survival and functional outcome between FTY720 treated mice and controls. Furthermore, there was no difference between groups in terms of brain edema formation, a critical component in the clinical course of ICH. Our results suggest that FTY720 treatment is not beneficial after ICH in the early phase.

Acute ICH is followed by a profound inflammatory response. The hematoma is surrounded by an inflammatory zone, characterized by BBB-breakdown and locally released chemokines which lead to the immigration of inflammatory cells from the blood stream into the perihematomal zone [19–22]. In experimental studies on ischemic stroke, the subpopulation of T-lymphocytes has been identified to play a pivotal role in the progression of the disease, and anti-inflammatory regimes targeting these cells were suggested to provide neuroprotection [7, 9, 23, 24]. FTY720, has therefore been studied as a therapeutic option in ischemic stroke in several experimental settings. With the exception of one, all of these studies reported FTY720 to be beneficial in terms of reducing infarct size and improving functional outcomes [5, 6, 8, 9, 25]. How these protective effects of FTY720 are mediated in detail is unknown. Peripheral immunomodulation as the underlying mechanism is supported by a recently published study of Kraft et al., in which FTY720 was tested in wild-type and lymphocyte-deficient RAG1−/− mice. Interestingly, in this study lymphocyte reduction in the cerebral vasculature of FTY720 treated ischemic mice attenuated microvascular thrombus formation and increased cerebral blood flow [26]. This modulation of local thrombo-inflammatory processes in the ischemic brain microvasculature might not be operative in ICH and is a possible explanation for the absence of a positive treatment effect in our study.

Our results stand in contrast to the work of Rolland et al., which reported FTY720 to be neuroprotective in experimental ICH in terms of improving functional outcomes and reducing brain edema formation [4]. The reasons for the divergent results are not entirely clear. Although significant, the reported data from Rolland et al. show rather small absolute and relative differences between groups concerning functional outcomes after 24 h, which tend to diminish after 72 h. This questions the translational relevance of the findings. Furthermore, previous studies have shown that the number of lymphocytes in the brain is markedly increased 3 days after ICH onset [25]. One would therefore expect a stable or increasing effect of an anti-inflammatory therapy regime in this time frame rather than a decreasing. In the work by Rolland et al., edema reduction in FTY720 treated animals was detected mainly in the basal ganglia of ipsilateral hemispheres, based on wet-dry measurements. However, the hematoma itself was not removed. Edema values might therefore have been influenced by differences in ICH volume (which can vary in the collagenase model and which was not assessed). Also in a recently published paper, which reports FTY720 to reduce edema formation and to improve neurological function as early as 3 days after ICH, the hematoma was not removed before brain water content was measured [27]. Surprisingly in this work absence of an effect of FTY720 on inflammation is stated. Instead the authors discuss that the prevention of BBB breakdown via S1P1 receptors may lead to decreased edema [28, 29]. In our study, we also investigated MMP-9 levels. MMP-9 is a proteolytic enzyme which is associated with BBB disruption and edema formation [28, 29]. FTY720 administration did not result in a protective effect regarding the BBB.

In an observational study of 23 patients with supratentorial ICH FTY720 reduced perihematomal edema and improved functional outcomes if administered within 72 h of symptom onset [30]. One should be careful to generalize these results since the sample size was small and the study was not randomized. Furthermore concerns were raised if edema measurements were accurate since time points of imaging varied between groups and different imaging modalities were used [31]. However, FTY720 was administered at later time points as in our study and for 3 consecutive days. Therefore we cannot exclude the possibility of a beneficial effect of FTY720 if given at later time points. ‬‬‬‬‬‬‬‬‬‬‬‬‬

Our study has several limitations. First, we used a single experimental model and a single species of animals only. However, we believe that the large number of mice analyzed allows us to derive a reliable conclusion from our work for the tested hypothesis. Second, our study lacks a positive control. However, the researchers who conducted the experiments have longstanding experience with this experimental model and were able to demonstrate the effects of anticoagulation and anticoagulation reversal on hemorrhage growth and functional outcome in several studies, bolstering the reliability of this model [10, 11, 14–16]. Lastly, we used only a single concentration of FTY720 with a single administration since we did not focus on analyzing long-term outcome in our mice. In studies showing a positive drug effect, FTY720 was administered once daily over 3 days [27, 32]. In one of these studies the 3-day regimen was compared to a single administration and did only reveal mild differences between groups [32]. Furthermore a single administration with the identical dosage as in our study was used in experimental ischemic stroke showing a positive treatment effect after 24 and 72 h [33, 34]. However, FTY720 might have the potential to positively influence regeneration after ICH if administered over several days. This needs to be investigated in future translational studies.

In conclusion, our study suggests FTY720 not to be beneficial in acute ICH. However, further studies are needed to test its effectiveness in long-term outcome and regeneration.

## Additional file

10.1186/s13231-016-0016-z Representative gel image of zymogram showing MMP-9 positive bands.
