# Supplementary figures and images for: Treatment with FTY720 has no beneficial effects on short-term outcome in an experimental model of intracerebral hemorrhage
Source: Exp Transl Stroke Med. 2016 Feb 18;8:1. doi: 10.1186/s13231-016-0016-z (PMC4758011; doi:10.1186/s13231-016-0016-z)

## Slide 1
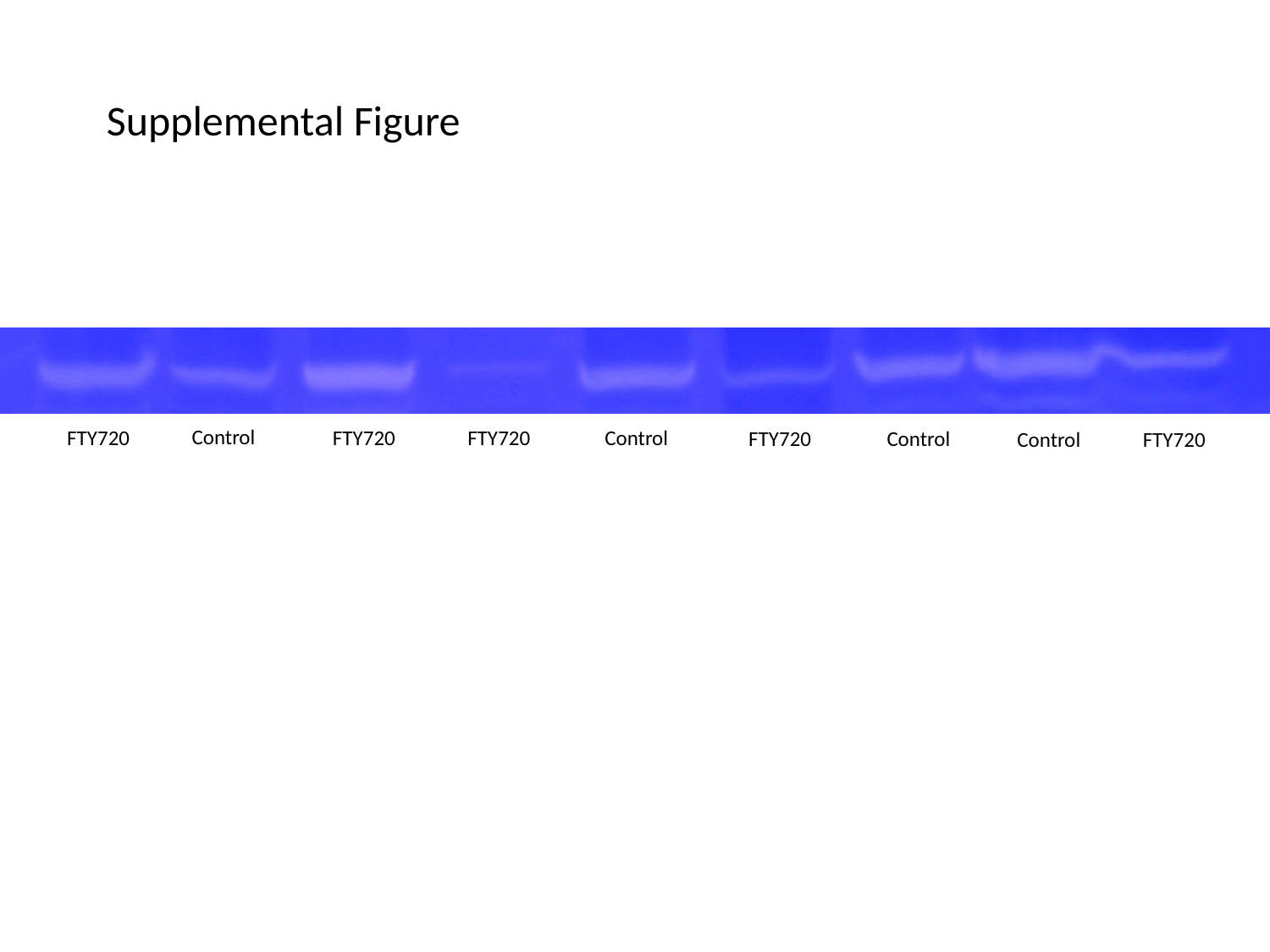

Supplemental Figure
Control
FTY720
Control
FTY720
FTY720
Control
FTY720
Control
FTY720

Supplement: Supplementary file 1 — 10.1186/s13231-016-0016-z Representative gel image of zymogram showing MMP-9 positive bands. [file 13231_2016_16_MOESM1_ESM.pptx]
